# Immune Thrombocytopenia (ITP) Secondary to Subclinical Hashimoto’s Thyroiditis: Role of Levothyroxine in Improving the Clinical Outcome of ITP

**DOI:** 10.1177/2324709616647085

**Published:** 2016-05-02

**Authors:** Hassan Tahir, Faizan Sheraz, Jahnavi Sagi, Vistasp Daruwalla

**Affiliations:** 1 Temple University/Conemaugh Memorial Hospital, Johnstown, PA, USA; 2 Social Security Hospital, Islamabad, Pakistan

**Keywords:** immune thrombocytopenia, subclinical Hashimoto’s thyroiditis, refractory ITP

## Abstract

Immune thrombocytopenia (ITP) is the most common cause of isolated thrombocytopenia in healthy people. ITP may rarely coexist with autoimmune thyroid disorders, which may indicate more complex defect in immune system. Primary ITP usually responds well to steroids and intravenous immunoglobulins. However, ITP may be difficult to treat when associated with thyroid autoimmune disorders. In such cases, treating the underlying thyroid disorder may significantly improve platelet count and can either cause remission of disease or improve response to standard ITP therapy. We report a case of 47-year-old male who was diagnosed with ITP and was also found to have subclinical Hashimoto’s thyroiditis. Treatment of subclinical hypothyroidism with levothyroxine in our patient significantly improved the platelets, thus successfully bringing the disease in remission.

## Introduction

Immune thrombocytopenia (ITP) is an autoimmune disorder, characterized by immune destruction of platelets leading to low platelet counts.^1^ The vast majority of ITP cases are idiopathic with no underlying cause, hence termed as primary ITP. Secondary ITP, on the other hand, is usually caused by a variety of conditions, which include hepatitis C virus (HCV), HIV, systemic lupus erythematosus, drugs, and malignancies. Other common causes of thrombocytopenia should always be taken into account and ruled out first before diagnosing a patient with ITP, as management strategy varies widely with different etiologies of thrombocytopenia. Symptoms of ITP vary from asymptomatic disease to life-threatening spontaneous bleeding. Association of Graves’ disease and Hashimoto’s thyroiditis with ITP has been documented in few reports and studies,^2^ but subclinical Hashimoto’s thyroiditis as the cause of secondary ITP is a very rare phenomenon. Recent studies have shown that treating thyroid autoimmune diseases improve the clinical course and overall outcome of ITP.^3,4^ We present a case of 47-year-old male who was admitted with severe ITP and was found to have subclinical Hashimoto’s thyroiditis. Treating subclinical hypothyroidism with levothyroxine in our patient significantly improved the platelet counts on the long run.

## Case Presentation

A 47-year-old male presented to the emergency department with the complaint of rash that he noticed 4 days ago. Rash started first on his back, which later spread to his abdomen and left arm. There was no itching or pain associated with the rash. The patient denied any fever, chills, sore throat, or recent sick contacts. Past medical history was significant for type 2 diabetes only for which he was taking metformin. The patient did not have any allergies, and he was not taking any medications other than metformin. On examination, vitals were stable but skin exam revealed petechial rash on back, abdomen, and extremities. There was no palpable lymphadenopathy or hepatosplenomegaly. Rest of the physical examination was unremarkable. In the emergency department, the patient’s complete blood count was done, which showed platelet count of 1000/µL only with normal white blood cell count (6.6 × 10^3^/µL) and hemoglobin (14.5 g/dL). Peripheral blood film showed thrombocytopenia with no shistocytes.

Differential diagnosis included other common causes of thrombocytopenia such as drugs, DIC (disseminated intravascular coagulation), viral infections, hypersplenism, nutrition deficiency (B_12_ and folate), and infiltrative marrow disorders. All common causes of thrombocytopenia were taken into account and ruled out before making the diagnosis of ITP. Isolated thrombocytopenia and normal peripheral blood film in the presence of unremarkable physical exam led to the presumptive diagnosis of ITP. As platelet counts were critically low (1000/µL), it was considered a medical emergency and the patient was treated immediately with ITP standard therapy, that is, intravenous immunoglobulins (IVIG) and steroids. All baseline investigations like basic metabolic profile, prothrombin time/international normalized ratio, partial thromboplastin time, and liver function test were normal. Vitamin B_12_ and folate levels were also within normal limits. These investigations helped in ruling out other important causes of thrombocytopenia. After starting ITP therapy, extensive workup was done to find any secondary cause of ITP. Urine drug screen, hepatitis panel, and HIV screening test were negative. Tests for autoimmune disorders like ANA and anti-dsDNA were also inconclusive. Thyroid antibodies were also ordered to screen for concurrent autoimmune thyroid disease in ITP, which came back positive for anti-TPO antibodies (462 IU/mL). Thyroid-stimulating hormone (TSH) was done subsequently, which was higher normal (4.52 µIU/mL), and free T4 and T3 were normal.

The patient was immediately treated with 0.5 g/kg/day of IVIG and high-dose steroids, which improved the platelet count to a safe level in 2 days, but it never returned to normal. The patient did not have any major bleed during the course of hospital stay. The patient was discharged with a maintenance dose of 40 mg of prednisone. The patient was not treated on levothyroxine for subclinical Hashimoto’s thyroiditis because there were no symptoms and hypothyroidism was subclinical. Despite steroid therapy, the patient’s platelet counts continued to drop, and couple of times it dropped to as low as 20 000. The steroid dose had to be increased to keep the platelets from dropping to critically low levels. The patient continued to have low platelets for months despite steroid therapy, and thrombocytopenia was considered refractory to steroids. The patient did not have any bleed, but he did have critically low platelets once in a while requiring increase in steroids. In the meantime, repeat TSH was done, which was slightly high and this time patient was started on levothyroxine. Interestingly, the patient’s platelet count started to increase, and in 3 weeks, his platelets returned to normal level. Prednisone was slowly tapered off and the patient continued on levothyroxine. Intriguingly, treatment of Hashimoto’s thyroiditis did improve the clinical course of ITP, which was considered refractory to steroids as the patient continued to have normal platelet counts on regular follow-up.

## Discussion

Immune thrombocytopenia is a destructive platelet disorder that can either be classified as primary ITP, which is idiopathic in origin, or secondary ITP, caused by variety of conditions like viruses, drugs, autoimmune disorders, infections, and malignancies. In ITP, there is autoimmune-mediated destruction of platelets directed against surface antigens, resulting in opsonization and destruction of platelets by reticuloendothelial system, particularly spleen.^5^ Both antibody-mediated destruction and suppressed platelet production contribute to reduced platelet life span.^6^ ITP is the most common cause of isolated thrombocytopenia in otherwise healthy people, with majority of patients being asymptomatic. Mild platelet type bleed like petechiae and purpura are also common, but life-threatening hemorrhage is rare and is usually seen with platelet counts of less than 10 000 to 20 000. Contrary to other causes of thrombocytopenia, ITP is the diagnosis of exclusion. Differential diagnosis of thrombocytopenia includes thrombocytopenic purpura, drug-induced thrombocytopenia, congenital thrombocytopenia, liver cirrhosis, viral infections, leukemia, and myelodysplasia. The most important diagnostic approach to ITP is excluding these important causes. Isolated thrombocytopenia with normal physical exam, laboratory data, and peripheral blood smear usually lead to presumptive diagnosis of ITP.^7^ Antiplatelet antibody testing for the diagnosis of ITP is not routinely recommended by the American Society of Hematology guidelines due to its low sensitivity and specificity and lack of correlation of antibodies with clinical outcomes.^7^ After diagnosing ITP, every effort should be made to rule out any secondary cause of ITP as treatment of underlying cause may improve platelet count. All patients should have peripheral blood smear, HCV, and HIV testing.^7^ Addition testing like bone marrow biopsy, thyroid profile, and coagulation and immunological studies are reserved for selected patients only. Indication of treatment of newly diagnosed ITP is platelet count less than 30 000 or count less than 50 000 with evidence of significant bleeding or risk of bleeding.^8^ Any life-threatening bleed should be treated with platelet transfusions, IVIG, and glucocorticoids. ITP-specific therapy is also recommended in patients with platelets below 30 000 without bleed.^8^ Treatment goal for ITP is not to bring platelet counts to normal but to maintain the platelet count at a level that successfully prevents spontaneous bleeding. Second-line therapies include splenectomy, rituximab, azathioprine, danazol, eltrombopag (thrombopoietin agonist). ITP secondary to autoimmune diseases is occasionally refractory to standard therapy; however, remission can be ensured if particular autoimmune disease is effectively treated.

Hashimoto’s thyroiditis is one of the most common causes of hypothyroidism and occurs in genetically predisposed individuals. Disorder is triggered by various environmental factors including infections and other autoimmune processes leading to production of auto-antibodies against thyroid gland resulting in hypothyroidism.^9^ Disease ranges from subclinical hypothyroidism to overt hypothyroidism, and rarely, it can be complicated by thyroid lymphoma. The patient may have features of hypothyroidism such as constipation, dry skin, weight gain, cold intolerance, and fatigue. However, in some patients, presentation may be subclinical, that is, without any symptoms and diagnosis is made by routine thyroid function testing. Hashimoto’s disease is usually diagnosed by high TSH and elevated antithyroid peroxidase (anti-TPO) and/or anti thyroglobin (anti-TG) antibodies. Subclinical hypothyroidism is characterized by upper normal or mildly high TSH with normal free T4 and T3. In such cases, mildly elevated TSH with high thyroid antibodies increases the risk of getting overt hypothyroidism in the future, and therefore, levothyroxine therapy is considered beneficial in such patients. All patients with TSH ≥10 mIU/L, or clinical features of hypothyroidism, should be treated.^10,11^ In patients with subclinical hypothyroidism and TSH <10 mIU/L, treatment should be considered with goiter, infertility, and high anti-TPO antibodies.^10^ Treatment of patients with anti-TPO Abs and TSH between 3 and 5 mIU/L is still controversial, though consensus can be made that these patients should be followed-up closely, almost every 6 to 12 months to monitor rise in TSH and hypothyroid symptoms. There is lack of evidence for the benefit of starting levothyroxine therapy in these patient groups.

The association between ITP and autoimmune thyroid disease has been described in various case reports.^12^ Since majority of medical literature regarding ITP and autoimmune thyroid disease comprise case reports and retrospective studies, the impact of treating thyroid disease on the clinical outcome of ITP is still debatable. ITP and thyroid disorder could indicate a much more significant defect in immune tolerance, thus making these patients more refractive to standard ITP therapy. Treating hypo-/hyperthyroidism in such patient groups has been shown in various reports to improve clinical outcome of ITP.^3,4^ As incidence of thyroid antibodies is high with ITP, it is reasonable to screen ITP patients with thyroid antibodies. If positive, patients should be further screened with TSH to detect subclinical thyroid disease. It has been reported that treatment of coexisting thyroid disease in ITP patients results in either remission of autoimmune thrombocytopenia or enhanced response to standard therapy of AITP.^3,4^ Whether subclinical Hashimoto’s thyroiditis with upper normal TSH should be treated or not is still debatable.

Our patient presented with petechial rash and was found to have very low platelets. Our differential diagnosis included drug-induced thrombocytopenia, viral infections, DIC, cirrhosis, and marrow infiltrative disorders. All baseline labs were ordered, which came back normal and helped rule out important differential diagnosis. Patient did not have any symptoms and labs showed isolated thrombocytopenia. Peripheral blood film was normal with no shistocytes. Presumptive diagnosis of ITP was made based on isolated thrombocytopenia with normal physical exam and laboratory investigations. Antiplatelet antibodies were not ordered due to low sensitivity and specificity of these tests. The patient was started on IVIG and steroids. In the meantime, further testing was done to find any secondary cause of ITP. Hepatitis panel and HIV screening tests were negative. Similarly, ANA and anti-dsDNA antibodies were also negative. Thyroid auto-antibodies were also ordered, which came back positive for anti-TPO. TSH was in high normal, and free T4 was within the normal range. A diagnosis of subclinical Hashimoto’s thyroiditis was made, as the patient did not have any symptoms of hypothyroidism. The patient was discharged on steroids but his platelet count continued to drop despite increasing the dose of steroids. There were a few times when platelets dropped to less than 30 000. Repeat TSH showed mildly high TSH with normal free T4, and this time, the patient was started on levothyroxine. Surprisingly, the patient’s platelet count started to improve and within a few weeks it returned to normal. Steroid was tapered off gradually and levothyroxine was continued. To our surprise, the patient did not develop thrombocytopenia again while on levothyroxine therapy. In our view, levothyroxine improved the overall clinical outcome of ITP.

## Conclusion

Immune thrombocytopenia can rarely coexist with Hashimoto’s thyroiditis. ITP in such cases might be refractory to standard first-line and second-line therapies due to much more significant defect in immune tolerance. Treating coexisting autoimmune disorder can improve the platelet count and therefore should be considered in such patients.

## Learning Points

1. Patients with ITP and Hashimoto’s thyroiditis can be more refractory to standard ITP treatment.
2. Treatment of Hashimoto’s thyroiditis with levothyroxine may either induce remission or enhance response to standard ITP therapy; therefore, treatment can be beneficial in such patients.
3. In patients with subclinical Hashimoto’s thyroiditis and TSH between 3 and 5 µIU/mL, decision can be made to start levothyroxine as treatment may improve the overall outcome.
4. Patients with ITP and no underlying cause may be screened with thyroid autoantibodies. If positive, TSH should be ordered and decision to treat should be made accordingly.
5. Further studies are needed to evaluate the effect of levothyroxine on the clinical outcome of ITP.
